# PIWI-interacting RNA-17458 is oncogenic and a potential therapeutic target in cervical cancer

**DOI:** 10.7150/jca.83446

**Published:** 2023-06-04

**Authors:** Lianqin Liu, Liu Li, Wufan Zu, Jiayu Jing, Guanjun Liu, Tingyi Sun, Qi Xie

**Affiliations:** 1 Department of Pathology, Henan Provincial People's Hospital; People's Hospital of Zhengzhou University; People's Hospital of Henan University, Zhengzhou, Henan, 450003, the People's Republic of China.; 2 Department of Nursing, Henan Provincial People's Hospital; People's Hospital of Zhengzhou University; People's Hospital of Henan University, Zhengzhou, Henan, 450003, the People's Republic of China.; 3 Department of Immunology, School of Basic Medical Science, Xinxiang Medical University, Xinxiang, Henan, 453003, the People's Republic of China.; 4 Department of Gynecology, Henan Provincial People's Hospital; People's Hospital of Zhengzhou University; People's Hospital of Henan University, Zhengzhou, Henan, 450003, the People's Republic of China.; 5 Internal Medicine Department of Oncology, Henan Provincial People's Hospital; People's Hospital of Zhengzhou University; People's Hospital of Henan University, Zhengzhou, Henan, 450003, the People's Republic of China.

**Keywords:** piRNA-17458, WTAP, m6A RNA methylation, cervical cancer, malignancy

## Abstract

Cervical cancer (CC) is one of the leading cancers among the female reproductive system. The piwi-interacting RNA (piRNA) function and biogenesis has been studied in various cancers, including CC. But the precise mechanism of piRNA in CC is still unknown. In our study, we found that piRNA-17458 was overexpressed in CC tissues and cells. piRNA-17458 mimic and inhibitor promoted and suppressed proliferation, migration and invasion ability of CC cells, respectively. We also demonstrated that piRNA-17458 mimic could contribute to tumor growth in mice xenograft models. Besides, we also found that the piRNA-17458 mimic could enhance mRNA N6-methyladenosine(m6A) levels and increase WTAP stability in CC cells, while the effects of the mimic was reversed by the WTAP knockdown. The results of dual luciferase reporter assay showed that WTAP was a direct target of piRNA-17458. Knockdown of WTAP attenuated proliferation, migration and invasion of CC cells in piRNA-17458 mimic group. Our finding not only demonstrates for the first time that piRNA-17458 is overexpressed in CC tissues and cells, but also shows that piRNA-17458 promotes tumorigenesis of CC in a WTAP-mediated m6A methylation manner.

## Introduction

Despite the widespread of human papilloma virus vaccination, CC is still one of the most common malignant tumors as a “mother killer”, threating women's health and their fertility 1, 2. Although great progress has been made in radio-chemotherapy and surgery, the high frequency of relapse, metastasis and drug resistance is an obstacle in clinical application 3, 4. Hence, an in-depth study into the mechanisms of CC is rather necessary to solve the above clinical problem.

Epigenetic regulation of mRNA performs an important role in the occurrence and development of cancer 5, 6. Modification of m6A modification, one of over 100 chemical modifications, means that mRNA can be modified by methylation at the N6 position of adenine and has been reported to play a crucial role in tumorigenesis and progression 7. The m6A modification is a dynamic process regulated by three kinds of genes, which are named methyltransferases, demethylases and m6A binding proteins 8-10. The methyltransferases, also called “writers”, catalyze the process of m6A modification and consist of WTAP, METTL3 and METTL14; the demethylases, also called “erasers”, catalyze m6A demethylation and consist of FTO and ALKBH5; the main function of m6A binding proteins is recognizing m6A modification process. There have been reports about the role of m6A RNA methylation regulators in tumor growth and progression of CC 11, 12. For example, Wang et al. demonstrated that METTL3 enhanced the HK2 stability via recruiting YTHDF1 and then contributed to the Warburg effect and tumorigenesis of CC 11. But the upstream regulatory mechanism of m6A methylation in CC have not been fully studied.

Recently, small non-coding RNAs play crucial roles in tumorigenesis and progression, suggesting that they may be as a molecular target for cancer diagnostics and therapeutics 13. These molecules mainly include microRNA (miRNA, length ~19-22 nt), piwi-interacting RNA (piRNA, length ~24-31 nt), small interfering RNA (siRNA, length ~20-24 nt) and so on. The piRNA was initially found to be expressed in germ cells and was later identified in cancer cells 14. It has been reported that piRNA is participated in cancer occurrence and development, and plays key roles in biological regulatory mechanisms, including silencing transposable elements, regulating mRNA stability and binding with many proteins 14, 15. But little is understood about the relationship in m6A and piRNA in CC. In our study, piRNA-17458 was identified with higher expression trends in CC tissues compared to normal tissues. We uncovered that piRNA-17458 may promote the progression of CC by increasing WTAP mRNA stability and m6A levels, suggesting that piRNA-17458 can be used as a potential prognostic biomarker in CC patients.

## Materials and Methods

### Tissue samples and and cell culture

5 pairs of paraffin-embedded CC samples and CC samples were collected from the Department of Pathology, Henan Provence People's Hospital. The inclusion criteria for the study were: (1) pathological diagnosis of CC without metastasis to regional lymph nodes or distant organs; (2) no any treatment before surgery; (3) matched healthy tissue samples can be obtained; (4) the patients ranged in age from 55 to 65 years.

The human cervical cancer cells (Caski and Siha cells) and the human normal cervical endometrial cells (End1/E6E7 and HUCEC cells) in study were purchased from Wuhan vector science Co.,Ltd (Wuhan, China). The End1/E6E7 and HUCEC cells were cultured in DMEM (HyClone, USA) with 10% FBS and 1% penicillin-streptomycin in a humidified atmosphere containing 5% CO_2_ at 37 °C. The Caski and Siha cells were cultured in RPMI-1640 (Hyclone, USA) plus 10% FBS and 1% penicillin-streptomycin in a humidified atmosphere containing 5% CO_2_ at 37 °C.

### RNA extraction, sequencing and data analysis

The RNeasy FFPE Kit was used to extract total RNA from the FFPE tissue according to manufacturer's instructions. RNA sequencing includes a ribosomal RNA depletion by ZapR v2 in the presence of mammalian-specific R-Probes. Subsequently, the libraries were sequenced on two lanes with 100 bp single-end sequencing cycles on an Illumina HiSeq 3000 (Illumina, Zurich, Switzerland). The DESeq2 was used to detect differential expression in RNA-seq data. DESeq2 normalized the counts of each gene employing a generalized linear model. Afterwards, DESeq2 used an empirical Bayes shrinkage to detect and correct for dispersion and log2-fold change estimates.

### Vectors and Transfection

piRNA-17458 mimic and piRNA-17458 inhibitor oligonucleotides, as same as the corresponding negative control, were synthesized from Ribobio (Guangzhou, China). The siRNA targeting WTAP sequences (si-WTAP#1 and si-WTAP#2, GenePharma, China) were used for silencing the expression of WTAP in this study. The two siRNA sequences used to target WTAP are as follows: si-WTAP#1: 5'-GGGUAUGCAGAGUACCAUUTTAAUGGUACUCUGCAUACCCTT-3' and si-WTAP#2: 5'-GCGAAGUGUCGAAUGCUUATTUAAGCAUUCGACACUUCGCTT-3'. Caski and Siha cells were seeded in the culture plate, and the transfection experiment was started on the second day after the transfection density was reached. According to the instructions, Lipofectamine 2000 (Invitrogen, Carlsbad, USA) and equal amounts of oligo fragments were diluted by Opti-MEM/Reduced serum medium (Thermo Fisher Scientific, Waltham, USA), respectively. The mixture was added into the culture plate after the two solutions were mixed.

### RNA Extraction and Quantitative PCR assay

Total RNA was extracted using Trizol reagent (Sigma, USA) and reverse transcribed into cDNA by Quantscript RT kit (abm, Richmond, BC, Canada) according to the manufactures' instructions. RNA expression was measured by qPCR by using the SYBR Premix Ex Taq™ (Takara, Japan). The relative expression of genes was calculated by using the 2-^ΔΔCt^ method with the normalization to GAPDH. Quantitative analysis of piRNAs was performed following the exactly same protocol with miRNA analysis. The primer sequences were synthesized from Ribobio (Guangzhou, China). U6 small nuclear RNA was used for normalization.

### MTT assay

The viability of CC cells was analyzed using MTT kit (Beyotime, China). CC cells were grown in 96-well plates (8×10^3^cells/well) for 24 h. Then, 90μl of fresh medium and 10μl of MTT reagent were added to each well to further culture the cells for 4 hours. Afterward, 110μl of Formazan solution was added and shaken for 10minutes. Finally, the absorbance was measured at 490nm using a microplate absorbance reader from Beyotime.

### Wound Healing Assay

A total of 3 × 10^5^ CC cells were seeded in a 6-well culture plate and incubated for 24 h followed by being scraped one parallel line with a sterile pipette tip, being washed thrice with PBS, and being cultured in RPMI-1640 containing 10% FBS. Images were taken under a microscope at 0 and 48 h after scratching (Olympus, Japan).

### The flow cytometry assay

Apoptosis rate was assayed with flow cytometry using the FITC Annexin V Apoptosis Detection Kit (cat. no. FXP018-100; both Beijing 4A Biotech Co., Ltd.). Cell cycle distribution was assayed with flow cytometry using Cell Cycle Analysis Kit (cat. no. FXP0311-100). The flow cytometry assay was performed using a previously described method 16.

### Transwell Invasion Assay

Transwell invasion assays were conducted in transwell chambers (Corning, USA) which was coated with Matrigel mix (BD Biosciences, USA) according to the manufacturer's protocol. After incubation for 48 h, the invaded cells were fixed with 4% parafor maldehyde for 15 min, and then stained with crystal violet (0.1%) for 15 min. Finally, an Olympus FSX100 microscope (Olympus, Japan) was used to photograph and count the stained cells.

### RNA m6A quantification

The m6A RNA methylation quantification kit (A-P-9005, A &D Technology, Beijing) was used to measure the m6A content in the total RNA. Briefly, 200 ng of RNA was coated on assay wells. Capture antibody solution and detection antibody solution were added to assay wells separately in a suitable diluted concentration following the manufacturer's instructions. The levels of M6A depend on the absorbance of each well at wavelength of 450 nm.

### Dual-luciferase reporter assay

WTAP was predicted to be a target gene of piRNA-17458 using miRanda, and their binding was verified using a luciferase reporter assay. The 3'-UTR of the WTAP was cloned into the pmirGLO vector, and a mutated sequence was generated and cloned into the vector. The reporter plasmids WTAP WT and MUT were constructed by GenePharma. At 48 hours after the transfection with piRNA-17458 mimic and WTAP-WT or WTAP -MUT, the luciferase activity was detected using a dual-luciferase reporter assay.

### Western Blot Analysis

CC cells were washed and lysed in RIPA lysis buffer. After that, the extracts were boiled for 5 min, separated on SDS-PAGE, and transferred to a PVDF membrane (Millipore, Germany). Subsequently, the PVDF membrane was incubated with the corresponding primary antibody at 4°C overnight. Then, the membrane was washed five times and incubated with a secondary antibody. Finally, bands were visualized by using an imaging system (Bio-Rad, USA). Target genes were normalized to corresponding β-actin using Image J software. The antibodies used in this study were as follows: WTAP (Proteintech, 60188-1-Ig) and β-actin (Proteintech, 66099-1-Ig).

### In vivo tumor xenograft model

Male BALB/c nude mice (3-4 weeks old) were purchased from Beijing Vital River Laboratory Animal Technology. All animal experiments were performed in strict accordance with the recommendations in the Guide for the Care and Use of Laboratory Animals and were approved by the Ethics Committee of the Henan Provincial People's Hospital. A total of 5×10^6^ Caski cells were subcutaneously injected into the left inguen of mice. When tumors were ~50 mm^3^±10% (day16). The mice were randomly divided into 4 groups and received piRNA-17458 mimic or piRNA-17458 inhibitor by intratumor injection for every 4 days for a total of five injections. On the 36th day, the tumor weight and lymph node metastasis of the nude mice were assessed after mice were euthanized.

### Statistical analysis

SPSS 19.0 was used for statistical analysis, and GraphPad Prism 5 software was used for graphing. Experimental data were all expressed as mean ± standard deviation (SD). Comparisons between two groups were performed using t-tests, and comparisons of multiple groups were performed using one-way analysis of variance (ANOVA). P<0.05 indicated a significant difference.

## Results

### piRNA-17458 is highly expressed in CC tissues and cells

**Figure 1:**
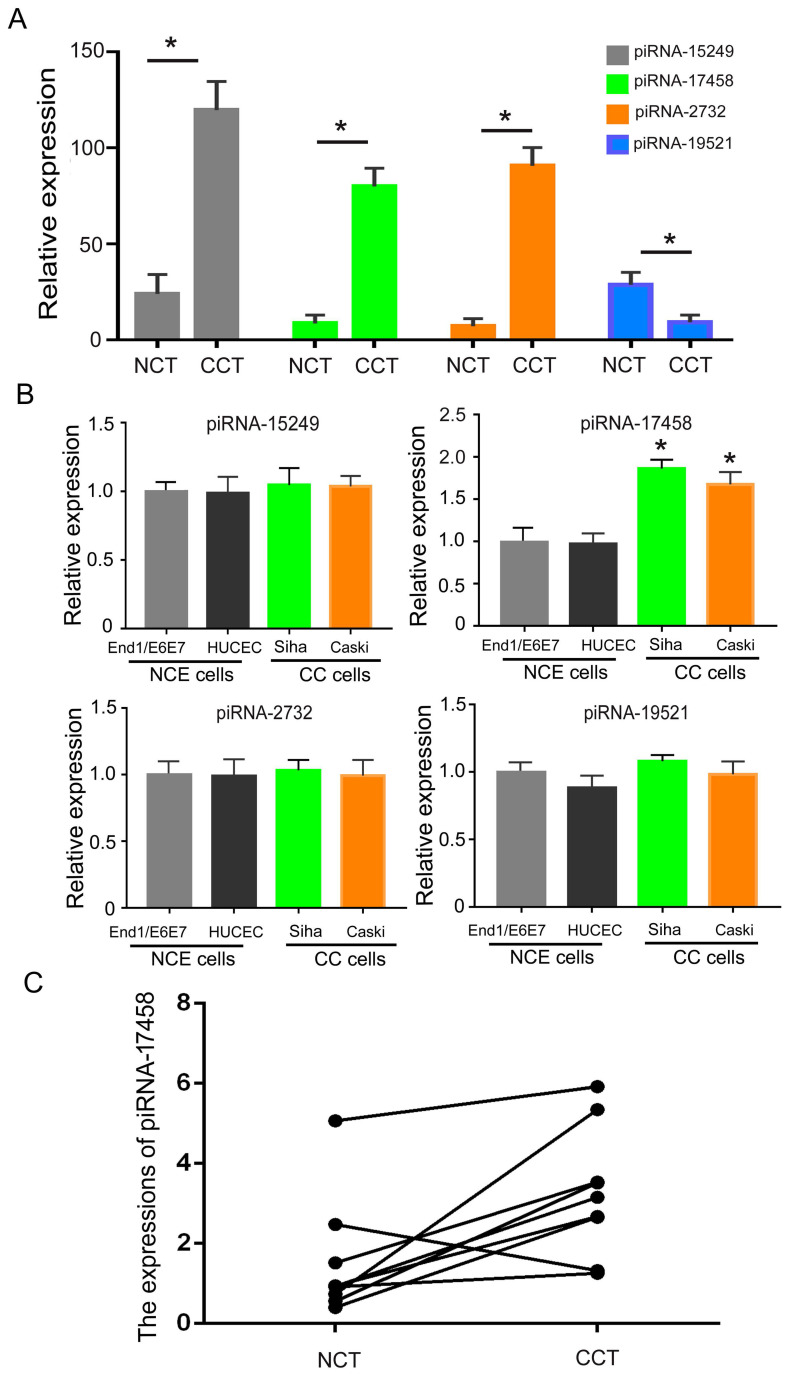
** piRNA-17458 is highly expressed in CC tissues and cells. (A)** The four piRNA expressions in CC tissues and NC tissues. **(B)** The four piRNA expressions in CC cells (Siha and Caski cells) and human normal cervical epithelial cells (HUCEC and End1/E6E7 cells). **(C)** The expression of piRNA-17458 in paraffin-embedded tissues of cervical carcinoma. *P < 0.05 vs. End1/E6E7 and HUCEC cells. ^#^*P* < 0.05 vs. End1/E6E7 and HUCEC cells. All experiments are expressed mean±SD. NCT: normal cervical tissue, CCT: cervical cancer tissue, NCE cells: normal cervical endometrial cells, CC cells: cervical cancer cells.

To analysis the expression of piRNAs in CC tissues, we identified differentially expressed piRNAs in CC tissues and matched controls by DESeq2 software. Table S1 showed expression levels of piRNAs in CC tissues and adjacent normal tissues. Then, we screened out four significantly different expressed piRNAs in two groups. As shown in Figure 1A, piRNA-15249, piRNA-17458 and piRNA-2732 were highly expressed in CC tissues, piRNA-19521 had a relatively low expression in CC tissues. Next, we validated the four piRNAs expressions in CC cells (Siha and Caski cells) and human normal cervical epithelial cells (HUCEC and End1/E6E7 cells). The qRT-PCR results showed that only piRNA-17458 expression had a statistically significant difference in CC cells and human normal cervical epithelial cells (Figure 1B). The expression of piRNA-17458 in cervical carcinoma was higher than that in normal cervical tissues in Figure 1C. These results suggested that piRNA-17458 might be a potential biomarker for CC diagnosis and prognosis.

### Overexpression of piRNA-17458 stimulates proliferation in vitro and tumor growth in vivo

**Figure 2:**
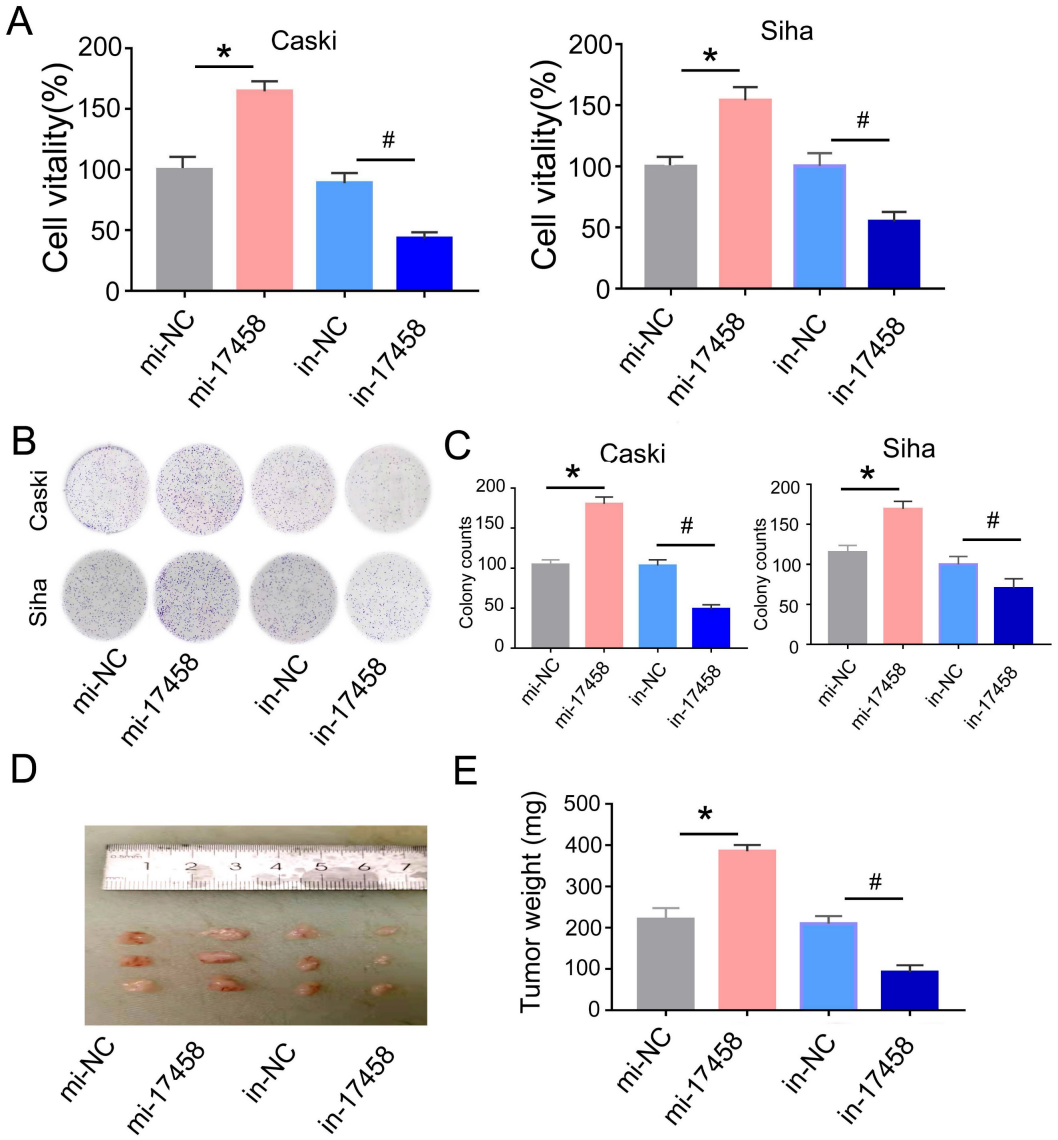
** Overexpression of piRNA-17458 stimulates proliferation in vitro and tumor growth in vivo. (A)** MTT assay showing the viability of cells after transfection with piRNA-17458 mimic or inhibitor.** (B and C)** A colony formation assay was performed to study the proliferation ability of cells after transfection with piRNA-17458 mimic or inhibitor. **(D and E)** Representative image of tumors were formed and tumor weight were evaluated in cells transfected with piRNA-17458 mimic or inhibitor. *P< 0.05 *vs.* mi-NC group, ^#^*P*<0.05 *vs.*in-NC group. mi-17458: piRNA-17458 mimic, in-17458: piRNA-17458 inhibitor, mi-NC: negative control mimic, in-NC: negative control inhibitor.

Next, we explored the regulative role of piRNA-17458 in CC. We transfected piRNA-17458 mimic or piRNA-17458 inhibitor into Caski and Siha cells, respectively. The qRT-PCR was performed to confirm the transfection efficiency (Figure S1). MTT results showed that piRNA-17458 mimic contributed to proliferation of Siha and Caski cells, and piRNA-17458 inhibitor suppressed cell proliferation activity (Figure 2A). Meanwhile, colony formation assays revealed that increased number of clones were found in piRNA-17458 mimic group, and reduced number of clones were found in piRNA-17458 inhibitor group (Figure 2B and C).

To evaluate the role of piRNA-17458 in tumor growth in vivo, Caski cells were injected subcutaneously into nude mice and tumor volume was calculated every four days. As shown in Figure 2D and E, piRNA-17458 mimic contributed to increased tumor growth and piRNA-17458 inhibitor suppressed tumor weight. In summary, these observations demonstrated that piRNA-17458 accelerated cancer proliferation in vitro and in vivo.

### piRNA-17458 induces S/G2 arrest and has no effect on apoptosis of CC cells

**Figure 3:**
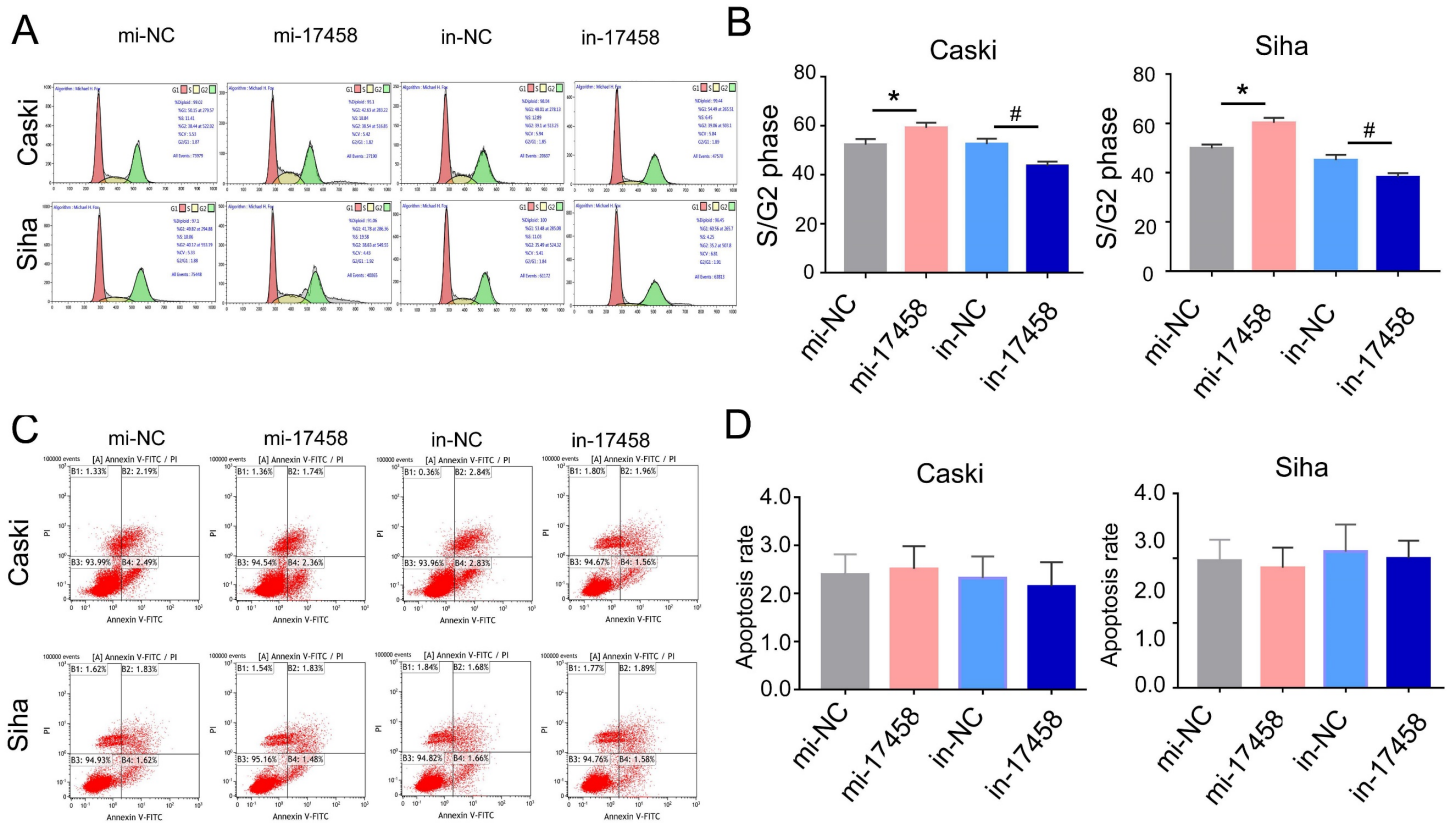
** piRNA-17458 induces S/G2 arrest and has no effect on apoptosis of CC cells. (A and B)** Effects of piRNA-17458 mimic or inhibitor on cell cycle distribution in Caski and Siha cells. **(C and D)** The proportion of apoptotic cells in Caski and Siha cells measured using flow cytometry. *P< 0.05 vs. mi-NC group, ^#^*P*<0.05 vs. in-NC group. mi-17458: piRNA-17458 mimic, in-17458: piRNA-17458 inhibitor, mi-NC: negative control mimic, in-NC: negative control inhibitor.

Usually, the cell can be regulated by cell cycle distribution and apoptosis signaling pathways. The flow cytometry analysis was performed to study the effects of piRNA-17458 on cell cycle progression and apoptosis. As shown in Figure 3A and B, the percentage of cells at S/G2 was significantly increased in piRNA-17458 mimic group compared to the control group. Flow cytometry also showed that piRNA-17458 had no effect on cell apoptosis (Figure 3C and D). These results confirmed that the growth promoting activity of CC cells transfected with piRNA-17458 mimic was achieved by cell cycle distribution.

### Overexpression of piRNA-17458 promotes migration and invasion of CC cells

**Figure 4:**
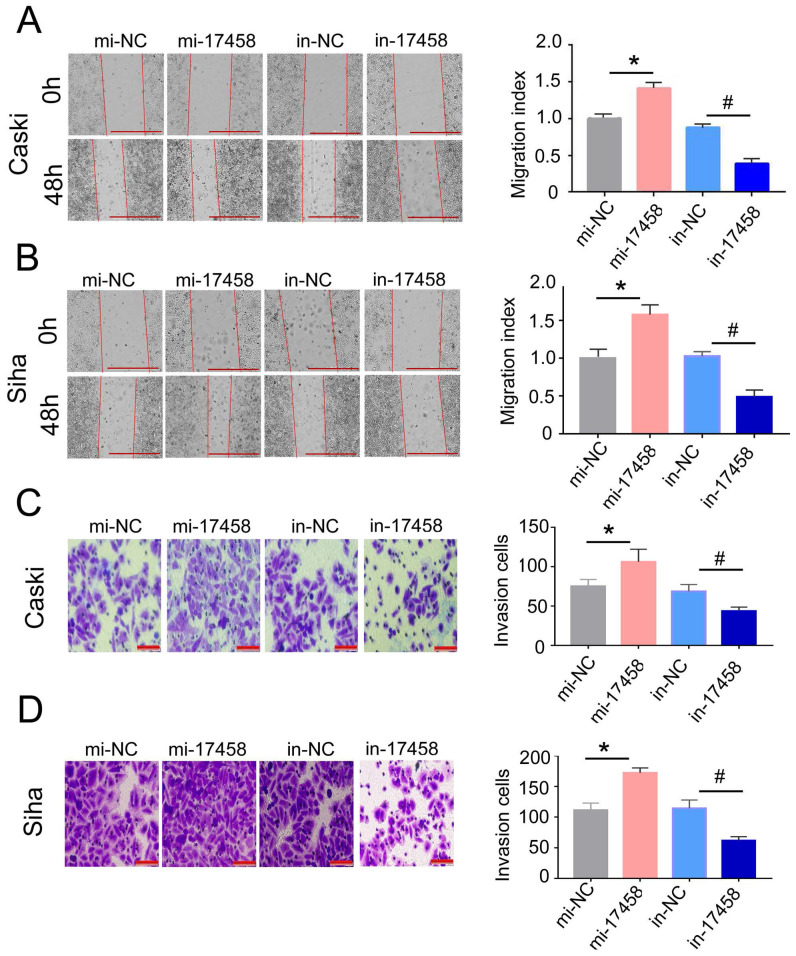
** Overexpression of piRNA-17458 promotes migration and invasion of CC cells. (A and B)** Wound healing assays were performed in Caski and Siha cells transfected with piRNA-17458 mimic or inhibitor. The migration ability was determined by measuring the distance from the boundary of the scratch to the cell-free space after 48 h. Scale bar = 1000 μm **(C and D)** The invasion ability of Caski and Siha cells measured using transwell assay. Scale bar = 50 μm *P < 0.05 vs. mi-NC group, ^#^*P* <0.05 vs. in-NC group. mi-17458: piRNA-17458 mimic, in-17458: piRNA-17458 inhibitor, mi-NC: negative control mimic, in-NC: negative control inhibitor.

To further illustrate that the role of piRNA-17458 in CC migration and invasion, the wound healing assay was done in piRNA-17458 mimic or inhibitor group. We found that a higher rate of healing in piRNA-17458 mimic group, whereas piRNA-17458 inhibitor group showed decreased healing (Figure 4A and B). Similarly, transwell assays showed that the invasion rate of CC cells in piRNA-17458 mimic group was increased, while knockdown of piRNA-17458 reduced the invasion rate of CC cells (Figure 4C and D). These data suggested that piRNA-17458 positively regulated migration and invasion in CC cells.

### piRNA-17458 elevates m6A RNA methylation levels in CC cells

**Figure 5:**
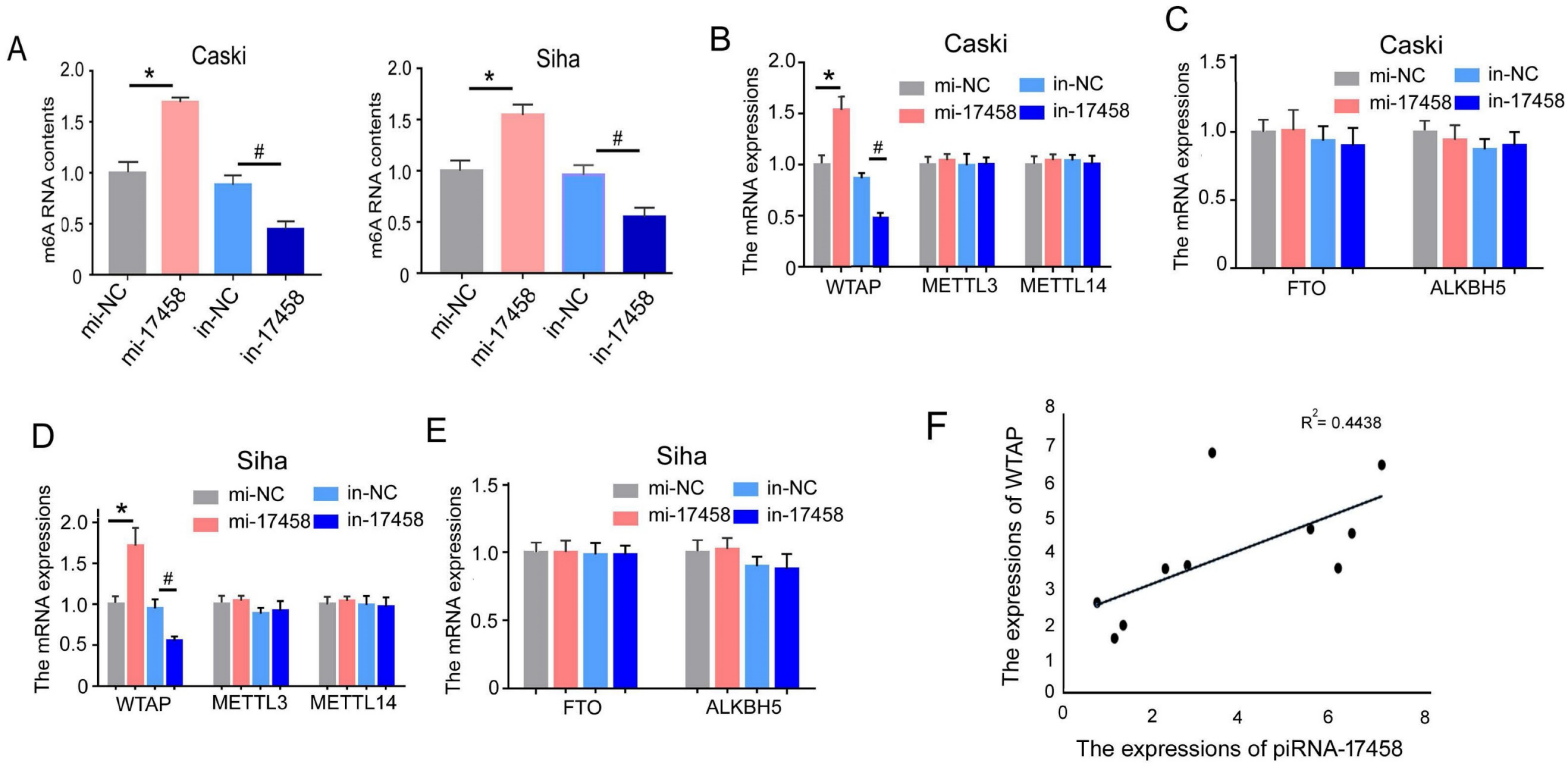
** piRNA-17458 elevates m6A RNA methylation levels in CC cells. (A)** The m6A content quantitative analysis in Caski and Siha cells transfected with piRNA-17458 knockdown or piRNA-17458 overexpression. **(B-E)** The qRT-PCR analysis showing the expressions of METTL3, METTL14, WTAP, FTO and ALKBH5 in CC cells. **(F)** The correlation of piRNA-17458 with WTAP expression in clinical FFPE specimens. *P < 0.05 vs. mi-NC group, ^#^*P* <0.05 vs. in-NC group. mi-17458: piRNA-17458 mimic, in-17458: piRNA-17458 inhibitor, mi-NC: negative control mimic, in-NC: negative control inhibitor.

To detect the effect of piRNA-17458 on m6A RNA methylation in CC cells, we analysed m6A levels in the total RNAs of CC cells with overexpression and knockdown of piRNA-17458. As shown in Figure 5A, we observed a dramatic increase in m6A levels in cells after piRNA-17458 overexpression, while piRNA-17458 knockdown transfection could reduce m6A levels. To further investigate the reason of altered expression of m6A levels in piRNA-17458 mimic or inhibitor group, we detected the expression levels of methyltransferase (METTL3, METTL14, and WTAP) and demethylase (FTO, ALKBH5) by qRT-PCR. These results showed that piRNA-17458 could increase the expression of WTAP mRNA and had no effect on the other four genes (Figure 5B-E). In clinical specimens, we found that the expression of piRNA-17458 showed a positive correlation with WTAP mRNA levels (Fig. 5F).

### piRNA-17458 targets 3'-UTR of WTAP and increase its stability in CC cells

**Figure 6:**
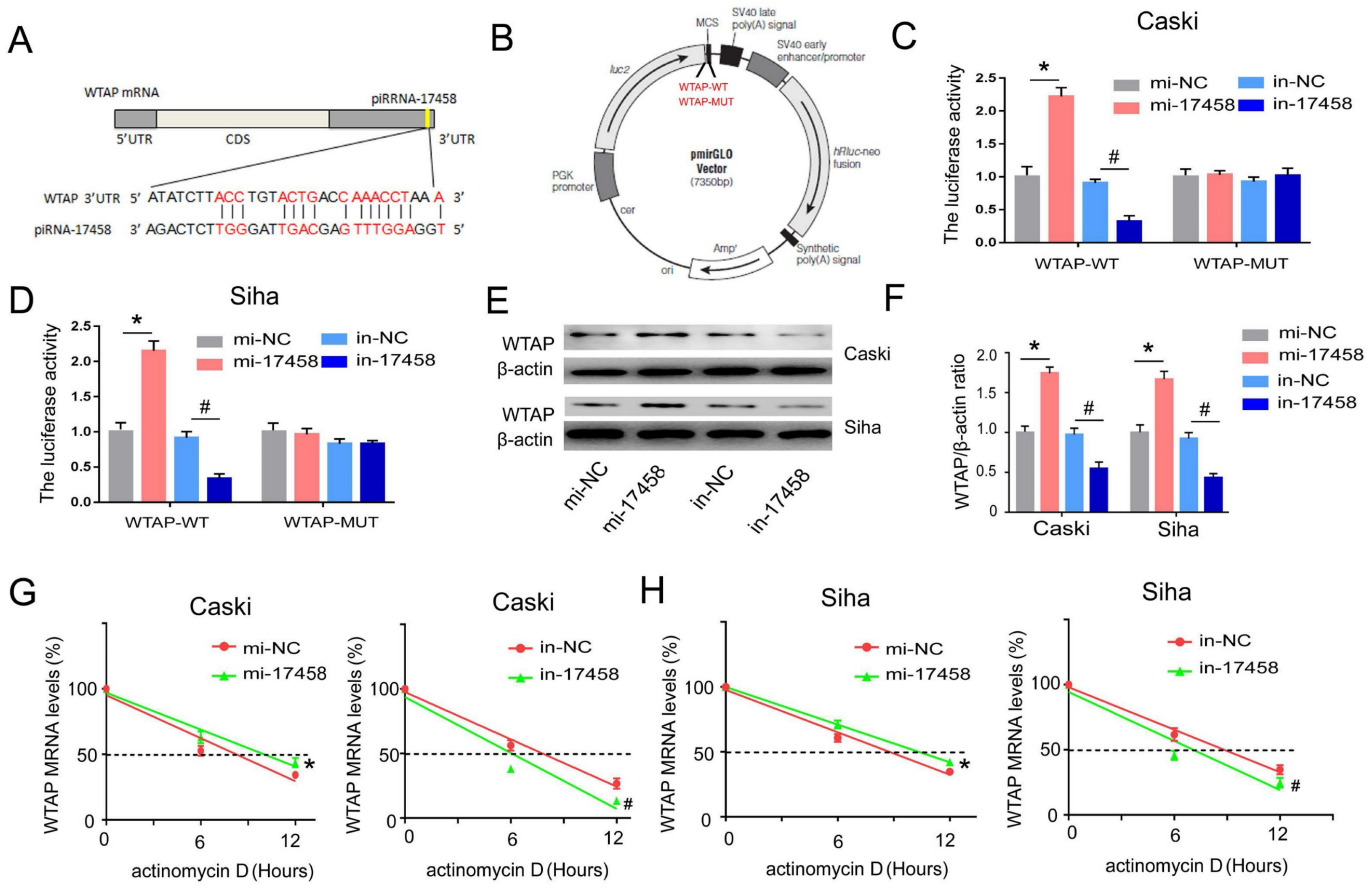
** piRNA-17458 targets 3'-UTR of WTAP and increase its stability in CC cells. (A)** The miRanda predicted the complementary sequence of WTAP as piRNA-17458.** (B)** The grammatic sketch presented the construction of WTAP-WT and WTAP-MUT. **(C and D)** Luciferase reporter assay experiment in Caski and Siha co-transfected with piRNA-17458 mimic or inhibitor and WTAP (MUT and WT).** (E and F)** The protein expressions of WTAP in Caski and Siha cells were detected by western blot. **(G and H)** RNA decay rate followed by RT-PCR assay showing WTAP mRNA half-lives. Data were detected at indicated timepoint with actinomycin D (Act D, 5 μg/ml) treatment. *P < 0.05 vs. mi-NC group, ^#^*P* <0.05 vs. in-NC group. mi-17458: piRNA-17458 mimic, in-17458: piRNA-17458 inhibitor, mi-NC: negative control mimic, in-NC: negative control inhibitor.

To determine whether piRNA-17458 had a putative binding site within WTAP mRNA, miRanda was used to predict target interaction between piRNA-17458 and WTAP. The miRanda predicted that 3'-UTR of WTAP had a sequence site binding to piRNA-17458 (Figure 6A). Then, vectors carrying WT- and MUT-WTAP 3'-UTR sequences were constructed (Figure 6B). The dual luciferase reporter gene assay showed that the relative luciferase activity was significantly increased in CC cells transfected with WT-WTAP 3'-UTR and piRNA-17458 mimic, while the relative luciferase activity was significantly decreased in CC cells transfected with WT-WTAP 3'-UTR and piRNA-17458 inhibitor. But there was no significant difference in CC cells transfected with the MUT-WTAP 3'-UTR and piRNA-17458 mimic or inhibitor (Figure 6C and D). Next, we explored the effect of piRNA-17458 on WTAP protein expression in CC cells. As shown in Figure 6E and F, the immunoblotting analysis also showed that piRNA-17458 increased the protein expression of WTAP. To further study the mechanism of piRNA-17458 regulating WTAP mRNA, RNA decay rate assay was performed. The half-life of WTAP mRNA was detected by actinomycin D. PiRNA-17458 prolonged the half-life of WTAP mRNA compared with the control group. While, knockdown of piRNA-17458 in cells led to shorter WTAP mRNA half-life compared to their control counterparts (Figure 6G and H). These results suggested that piRNA-17458 directly interacts with 3'-UTR of WTAP and enhances WTAP mRNA stability.

### piRNA-17458 contributes to CC proliferation via targeting WTAP

**Figure 7:**
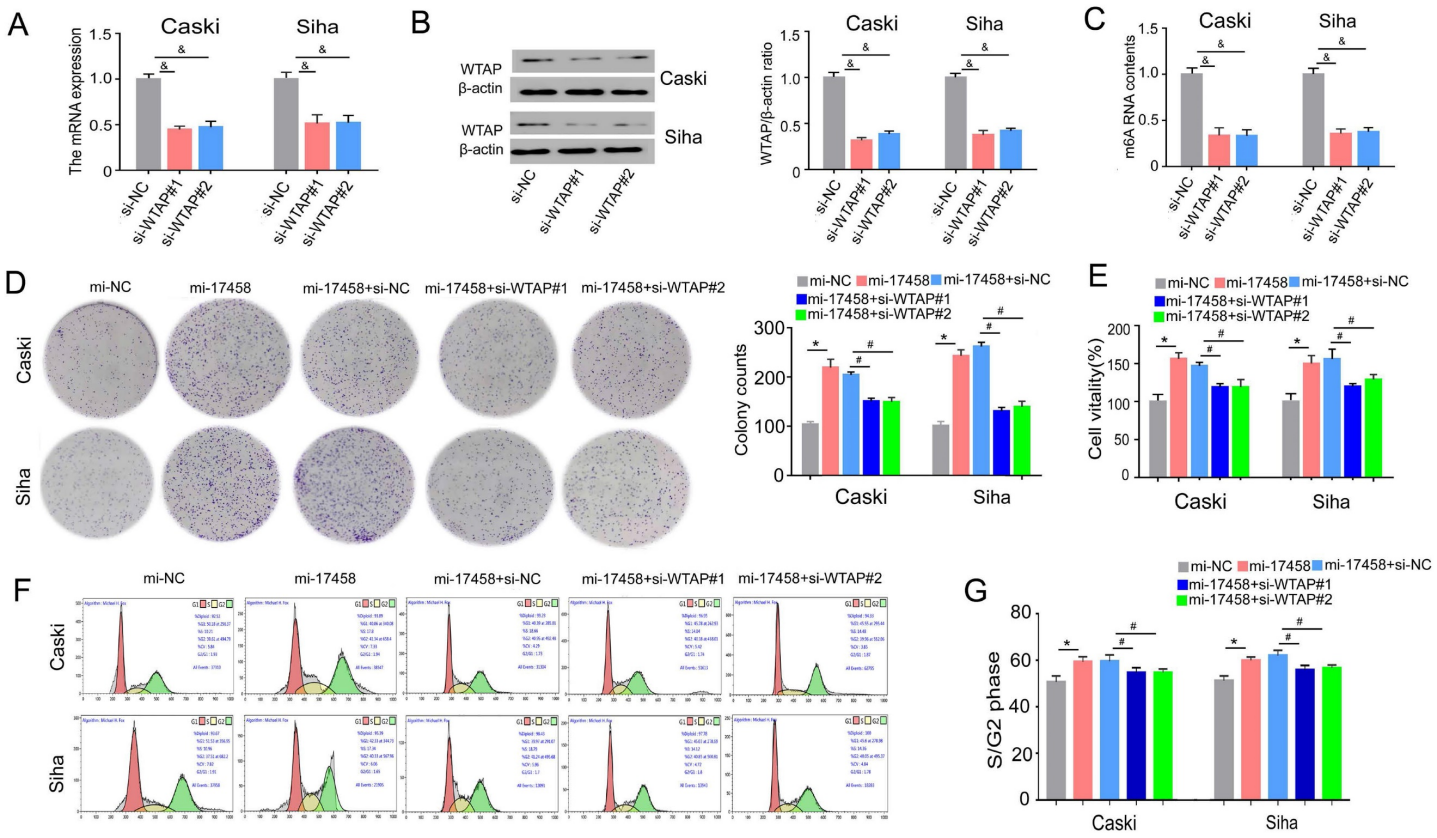
** piRNA-17458 contributes to CC proliferation via targeting WTAP. (A and B)** The qRT-PCR and western blot assay were used to test effect of siRNA transfection on WTAP.** (C)** The m6A content quantitative analysis in Caski and Siha cells transfected with WTAP knockdown.** (D)** Colony formation assay indicated the clones' number of CC cells. **(E)** CCK-8 assay illustrated the proliferative ability in CC cells with piRNA-17458 overexpression and WTAP knockdown. **(F and G)** Effects of piRNA-17458 overexpression and WTAP knockdown on cell cycle distribution in Caski and Siha cells. ^&^*P* < 0.05 vs. si-NC group. ^*^P < 0.05 vs. mi-NC group. ^#^*P* < 0.05 vs. mi-17458+si-NC group. mi-17458: piRNA-17458 mimic, in-17458: piRNA-17458 inhibitor, mi-NC: negative control mimic, in-NC: negative control inhibitor, si-WTAP, WTAP siRNA.

To study the role of WTAP in piRNA-17458-mediated progression. Effect of siRNA transfection on WTAP was tested by qRT-PCR and western blot assay (Figure 7A and B). We also validated that WTAP siRNA could decrease the m6A levels in the two cells (Figure 7C). The results of colony formation and MTT assay showed that the number of cell colonies formed and viability in the two piRNA-17458-overexpressing cell lines was partly reversed after transfecting the cell lines with WTAP siRNA (Figure 7D and E). Next, we analyzed how WTAP affected the cell cycle in piRNA-17458-overexpressing cells. Figure 7F and G shows representative histograms of cell cycle distribution in the two cells transfected with WTAP siRNA or piRNA-17458 mimic. These results showed that piRNA-17458 overexpression resulted in an increased proportion of cells in S/G2, which was reduced after transfecting with WTAP siRNA. Hence, we conclude that piRNA-17458-mediated proliferating effects is dependent on WTAP.

### piRNA-17458 binds to WTAP to promote migration and invasion of CC cells

**Figure 8:**
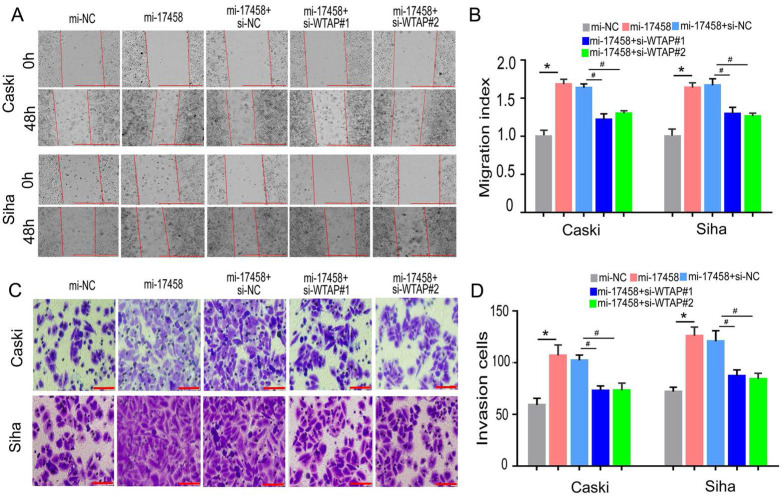
**piRNA-17458 binds to WTAP to promote migration and invasion of CC cells. (A and B)** The effect of piRNA-17458 overexpression and WTAP knockdown on cell migration ability detected by the wound-healing experiment after 48 h transfection. Scale bar = 1000 μm **(C and D)** Transwell assays detecting invasion of CC cells. Scale bar = 50 μm ^&^*P* < 0.05 vs. si-NC group. ^*^P < 0.05 vs. mi-NC group. ^#^*P* < 0.05 vs. mi-17458+si-NC group. mi-17458: piRNA-17458 mimic, in-17458: piRNA-17458 inhibitor, mi-NC: negative control mimic, in-NC: negative control inhibitor, si-WTAP, WTAP siRNA.

To confirm that piRNA-17458 could promote migration and invasion of CC through WTAP/m6A signaling axis, the wound healing and transwell assay were performed to study the migration and invasion abilities. Wound healing assay showed that cell migration rate in piRNA-17458 mimic group was upregulated compared with piRNA-17458 mimic+WTAP siRNA group (Figure 8A and B). Meanwhile, transwell cell assays showed that the invasion capacity of the two cells overexpressing piRNA-17458 was significantly attenuated after transfecting with WTAP siRNA (Figure 8C and D). Therefore, these results indicated that piRNA-17458 promotes migration and invasion of CC cells in a WTAP-dependent m6A methylome manner.

## Discussion

In this study, we have confirmed that piRNA-17458 was an oncogenic mediator in CC. The piRNA-17458 expreesion was significantly elevated in CC tissue samples and cell lines when compared with matched controls. PiRNA-17458 increased the percentage of S/G2 phase in the cell cycle, and also induced proliferation and elevated colony formation ability of Caski and Siha cells. Additionally, we also validated that piRNA-17458 promoted invasion and migration of CC cells. Using mice xenograft models, we have further demonstrated that piRNA-17458 promoted the growth of CC in vivo. The m6A and WTAP levels were increased in the piRNA-17458 overexpression group. WTAP knockdown could reverse the effect of piRNA-17458 on CC cells, suggesting that piRNA-17458 promotes tumorigenesis and metastasis of CC via WTAP-mediated m6A methylation.

Although there are a lot of reports about the various biological functions of piRNAs in occurrence and development of cancer 17-20, the relationship between piRNAs and CC is not well known. It has been shown that piRNA-36712 was low expressed in breast cancer than in matched controls and its overexpression restrains progression and chemoresistance of CC cells by interacting with SEPW1P RNAs 21. By piRNA expression profile analysis, Bartos et al. report that piRNA-9491 and piRNA-12488 were significantly dysregulated in glioblastoma cells and could reduce the ability to form colonies in vitro 17. Here, we showed that piRNA-17458 was significantly overexpressed in CC tissues and cells when compared with that in matched controls. The piRNA-17458 overexpression promoted proliferation, invasion and migration of CC cells. In Caski-xenograft models, piRNA-17458 promoted tumor formation and growth in nude mice. In summary, piRNA-17458 was elaborated as tumor promoting factor that promoted the malignant biological phenotype in CC. Interestingly, we also find that the changes of m6A RNA levels in piRNA-17458 overexpression and knockdown groups.

In our study, the mechanism of PiRNA-17458 in tumor is related to methylation, and some mechanisms of other piRNAs have been reported in the literature. Histone modification is the process of methylation, acetylation, phosphorylation and ubiquitination of histone under the action of related enzymes. These modifications can affect the transcription activity of genes 22. Different types of piRNAs can recruit corresponding histone-modifying enzymes to produce different effects. One study demonstrated that pi-sno75 binds to PIWIL1/4 and exerts an inhibitory effect in breast cancer by recruiting the MLL3/hCOMPASS complex to induce H3K4 methylation of TRAIL, upregulating the expression of the pro-apoptotic protein TRAIL 23. Some studies have shown that the imbalance of piRNA in tumors is closely related to the changes of DNA methylation. PiRNA-651 recruits DNMT1 to the promoter region of tumor suppressor gene PTEN via PIWIL2, thereby reducing its expression levels and promoting proliferation and invasion of breast cancer cells 24.

As the main way of RNA modification, m6A methylation is a dynamic and reversible process with various biological functions in cancer. About one-third of mRNAs are modified by m6A, regulate the occurrence, development and clinical prognosis of multiple cancers 25, 26. The disturbance of m6A levels has been reported to play a vital role in cancer progression and chemotherapy 27. Nie et al. have demonstrated that ALKBH5-HOXA10 loop contributes to cisplatin resistance of epithelial ovarian cancer in JAK2 m6A demethylation-dependent manner 28. Interestingly, by mass spectrometry, Huang et al. showed that m6A RNA methylation levels are markedly higher in circulating tumor cells compared to whole blood cells, suggesting that m6A RNA methylation levels can monitor cancer occurrence and development 29. It has also been reported that m6A RNA methylation plays a crucial role in tumorigenesis and progression of CC 30. For example, Li et al. have demonstrated that m6A contributes to the glycolysis of CC cells by targeting a complementary sequence in the 3'UTR of PDK4 and enhancing the stability of its mRNA 30. But it is not entirely clear whether there exists a relationship between piRNAs and m6A in CC. In this content, we found that piR-17458 increased m6A RNA methylation levels in CC cells.

In order to further explore mechanism underlying piR-17458-induced increase in m6A RNA methylation levels, we detected the expressions of m6A regulatory genes by qRT-PCR in CC cells. The results showed that piR-17458 increased WTAP mRNA expression by targeting 3'-UTR of WTAP in CC cells. In fact, WTAP is widely expressed in many types of tumors and is involved in numerous biological processes. The high expression of WTAP is significantly associated with poor prognosis in gastric cancer patients 31. Besides, WTAP acts as a tumor suppressor gene by methylating 3'-UTR of CAV-1 and then activating NF-κB signaling pathway in endometrial cancer 32. But there have been no reports about the role of WTAP in CC. In this study, after WTAP was knocked down, m6A RNA methylation levels and cancer progression were inhibited in piR-17458 overexpression group, suggesting that piR-17458 promotes CC progression by increasing WTAP expression.

## Conclusions

**Figure 9:**
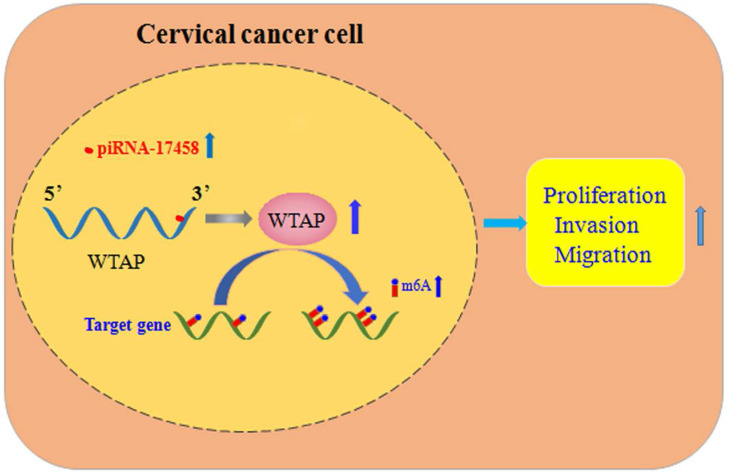
** piRNA-17458 promotes tumorigenesis and metastasis of CC via WTAP-mediated m6A methylation.** piRNA-17458 expression was significantly elevated in CC cells. piR-17458 enhanced WTAP stability and by the complementary sequences of the 3' UTR, and then elevated the global m6A levels, consequently promoting CC malignancy.

In summary, piRNA-17458 enhances the WTAP stability, and then increases its expression by the complementary sequences of the 3' UTR, and finally elevates the global m6A levels, which consequently promotes CC malignancy (Figure 9). Our present study reveals that piR-17458 is initially characterized in CC as a novel tumor promoting factor that stimulates tumor growth in vivo and in vitro, suggesting that targeting piR-17458 can be used as a novel therapeutic strategy for the CC treatment.

## Supplementary Material

Supplementary figure and table.
