# Supplementary material for: PIWI-interacting RNA-17458 is oncogenic and a potential therapeutic target in cervical cancer
Source: J Cancer. 2023 Jun 4;14(9):1648–59. doi: 10.7150/jca.83446 (PMC10266240; doi:10.7150/jca.83446)
Supplement: Supplementary file 1 — Supplementary figure and table. [file jcav14p1648s1.pdf]

PIWI-interacting RNA-17458 is oncogenic and a potential therapeutic target in cervical cancer

Lianqin Liu[1], Liu Li[2], Wufan Zu[3], Jiayu Jing[4], Guanjun Liu[5], Tingyi Sun [1,*] and Qi Xie [1,*]

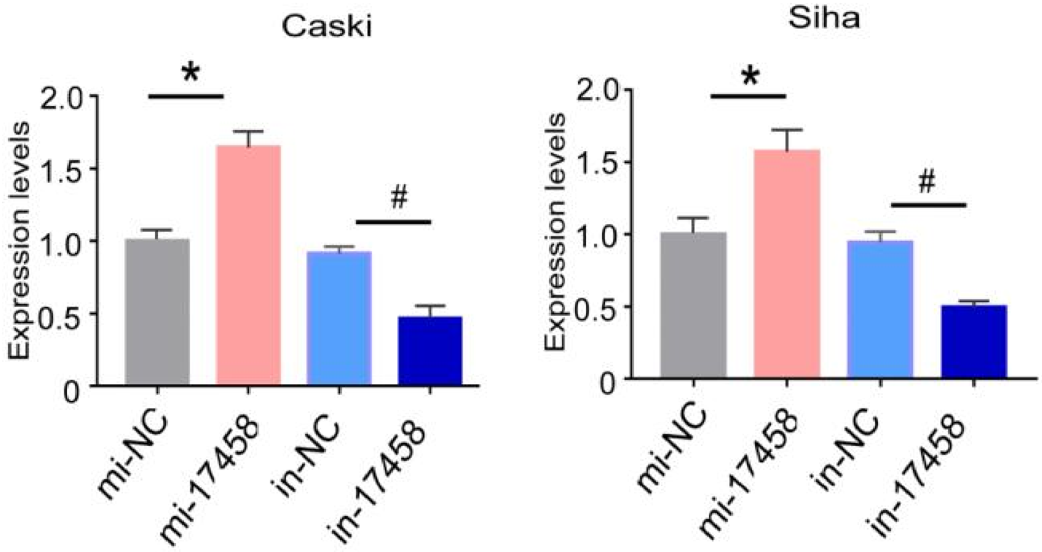

Figure S1. The piRNA-17458 mimic increased the expression of piRNA-17458 and inhibitor decreased the expression of piRNA-17458. The qRT-PCR was performed to confirm the transfection efficiency of piRNA-17458 mimic and inhibitor.

Table S1. The expressions of piRNAs in normal cervical and cervical cancer tissues

miRNA ID	Expression(N)	Expression(C)	log2FoldChange	Pvalue
PiRNA-17458	3.591276773	89.00719401	4.631353199	0.000555042
piRNA-15249	13.99510914	134.7615308	3.267414078	0.001214162
piRNA-02732	3.969526559	101.5754157	4.67744051	0.002303864

piRNA-19521	20.38743099	1.907349541	-3.418038832	0.004365726
piRNA-20657	90.7446541	8.912674627	-3.347882311	0.006303638
piRNA-20497	110.8629695	7.839784357	-3.821819774	0.006519503
piRNA-14633	4.227632893	93.78660601	4.47145989	0.007134486
piRNA-13350	22.30916272	217.3367918	3.284224147	0.012559204
piRNA-20809	7.593839715	89.95989063	3.566380454	0.016841762
piRNA-12721	3.213349442	52.10716689	4.019332033	0.018558002
piRNA-05446	4.877887489	68.86431469	3.819428189	0.019409965
piRNA-11186	4.226927034	67.00120559	3.986505941	0.019702521
piRNA-21190	0.155517538	2.141022444	3.783150733	0.02266689
piRNA-19308	6.922823213	0.485703628	-3.833212339	0.022707826
piRNA-20498	6.310144694	0.450579416	-3.807819774	0.023522786
piRNA-16677	221.524262	42.98837753	-2.365446149	0.02457841
piRNA-15151	12.55307143	180.3762635	3.844897195	0.024861756
piRNA-01356	0.215262739	2.917013589	3.760321582	0.024967848
piRNA-05076	3.185450007	0.245476716	-3.697839105	0.025850395
piRNA-14879	0.62145089	8.400779242	3.756810864	0.027476573
piRNA-19420	12.70669967	1.921301373	-2.725433624	0.028445182
piRNA-01089	12.09051485	137.0299765	3.502543945	0.030261238
piRNA-01312	55.39144634	413.9579756	2.9017492	0.036060103
piRNA-10756	0.450064138	5.248186472	3.543616463	0.038162246
piRNA-19825	13.4028257	82.26440205	2.61773108	0.041519411

piRNA-04640	3.66618448	39.65976504	3.435324842	0.044092006
piRNA-20381	0.756895332	6.658669385	3.137068194	0.045754188
piRNA-01205	9.496368543	47.76573066	2.330528103	0.047016968
piRNA-05019	29.14619298	148.2848203	2.34699156	0.049319469
piRNA-20793	2.751642265	0.276971863	-3.312481591	0.051109929
piRNA-06426	25.69994686	108.5906537	2.079062656	0.051848767
piRNA-01152	8.869191894	35.45754171	1.999217951	0.054962025
piRNA-04153	1.552717981	6.702953718	2.110001155	0.058150712
piRNA-18569	8.597054076	62.95212841	2.87234087	0.06235807
piRNA-20500	32.3994335	171.1607213	2.401311171	0.065141908
piRNA-09921	0.30759696	2.752597361	3.161680444	0.066207994
piRNA-01159	18.44971119	85.38717317	2.210421133	0.067217622
piRNA-20388	8.107310472	2.111779308	-1.940764319	0.068030277
piRNA-08113	2.254248303	0.262734532	-3.100968697	0.069604835
piRNA-16985	0.295458728	2.574490518	3.123258432	0.069755798
piRNA-04309	19.98634113	104.0136598	2.379686716	0.077249172
piRNA-17094	6.565401886	51.00181711	2.957593419	0.07999813
piRNA-21214	13.0838236	2.582454877	-2.340969167	0.081200562
piRNA-05271	48.27591942	144.6360258	1.583051301	0.081692976
piRNA-14620	13.22317922	59.06427865	2.159216788	0.082427186
piRNA-00823	31.08497235	7.742796788	-2.005290612	0.086044301
piRNA-08367	0.262101094	2.033044647	2.955446619	0.087048574

piRNA-11187	1.496814484	0.196433162	-2.929785009	0.087210305
piRNA-16658	72.81007708	258.2484395	1.82654959	0.088622756
piRNA-01040	18.99787604	60.52751589	1.671753009	0.092498104
piRNA-19912	35.00489162	124.8825825	1.834943832	0.095403607
piRNA-04307	25.56407542	8.613593695	-1.569430671	0.097462796
piRNA-13114	0.246145004	1.793984543	2.865587096	0.097533807
piRNA-00765	4.664957417	0.927561555	-2.330348982	0.09847727
piRNA-01633	2.773601697	0.39118387	-2.825841836	0.102342199
piRNA-15026	16.57777029	63.72273528	1.942558218	0.105581661
piRNA-14629	0.229610225	1.555503128	2.760122499	0.110981383
piRNA-00775	0.14671367	0.951276883	2.696862021	0.118870431
piRNA-05177	0.217809229	1.384369939	2.668092532	0.123747782
piRNA-06230	1.910692337	0.305669395	-2.644051476	0.127034173
piRNA-03985	0.212383059	1.317714101	2.633296791	0.128831254
piRNA-01847	0.212383059	1.317714101	2.633296791	0.128831254
piRNA-02528	10.87604397	66.55007393	2.61328638	0.129636276
piRNA-15254	1.848358335	6.446492025	1.802269832	0.130899228
piRNA-20499	20.03554702	6.601590977	-1.60167624	0.132952331
piRNA-12852	0.204021953	1.220540699	2.580724099	0.136776831
piRNA-20326	1.08472319	0.185908156	-2.544664961	0.141369162
piRNA-01346	15.93558107	6.559435387	-1.280608083	0.141778852
piRNA-01169	41.31368704	16.48688722	-1.32530078	0.148726724

piRNA-17178	3.00357407	0.6357196	-2.240217767	0.152348746
piRNA-19921	0.193908567	1.0811724	2.479148137	0.153043863
piRNA-08683	0.193908567	1.0811724	2.479148137	0.153043863
piRNA-17868	0.193908567	1.0811724	2.479148137	0.153043863
piRNA-14635	0.193908567	1.0811724	2.479148137	0.153043863
piRNA-11374	1.000525214	0.182253276	-2.456740874	0.156108945
piRNA-05023	0.18718718	1.008625006	2.429836358	0.161377644
piRNA-20439	1.401877891	0.260393408	-2.428595859	0.161574867
piRNA-19673	0.199993716	1.064467823	2.412105764	0.164450281
piRNA-01029	0.199993716	1.064467823	2.412105764	0.164450281
piRNA-02468	4.180622051	1.12732025	-1.890820207	0.166164673
piRNA-17791	4.790077873	16.57935051	1.791266475	0.169246415
piRNA-00794	0.746100073	0.146720285	-2.3463008	0.175963877
piRNA-09441	1.21781933	0.244234984	-2.31795835	0.181533809
piRNA-01170	14.09160273	49.71508586	1.818847991	0.182514005
piRNA-09294	5.689309306	2.142653254	-1.40885512	0.184344758
piRNA-00796	2.718442736	10.63992538	1.96863569	0.185387426
piRNA-16745	5.862714204	16.30695003	1.475846338	0.199978433
piRNA-01450	1.158135794	0.248435569	-2.220860775	0.200306705
piRNA-19168	40.59630018	15.06588837	-1.430062506	0.208415808
piRNA-20496	29.42786457	87.41319733	1.570668252	0.208831124
piRNA-08488	6.547468512	2.446599534	-1.42015924	0.209899336

piRNA-20391	2.964024158	9.456695693	1.673778967	0.213487973
piRNA-14637	0.682720165	0.156274114	-2.127215544	0.220114882
piRNA-00302	0.924484317	0.212669038	-2.120038833	0.220990451
piRNA-11188	0.924484317	0.212669038	-2.120038833	0.220990451
piRNA-18399	0.981438535	3.379191884	1.783708452	0.231266133
piRNA-09642	0.154855344	0.64623424	2.061136025	0.232644696
piRNA-09237	0.150999911	0.614048669	2.023805305	0.240710311
piRNA-18970	0.150999911	0.614048669	2.023805305	0.240710311
piRNA-03676	0.150999911	0.614048669	2.023805305	0.240710311
piRNA-18811	0.150999911	0.614048669	2.023805305	0.240710311
piRNA-14188	0.150999911	0.614048669	2.023805305	0.240710311
piRNA-09894	0.150999911	0.614048669	2.023805305	0.240710311
piRNA-12666	0.775996954	0.193661304	-2.002515279	0.246642648
piRNA-09265	0.779339176	0.195363699	-1.996088915	0.248098324
piRNA-20008	0.842614109	0.212314413	-1.988669763	0.249875066
piRNA-19824	151.0678125	51.82857939	-1.543376548	0.252285551
piRNA-20485	33.17600617	14.2716286	-1.216990244	0.252695968
piRNA-18780	8.358468802	2.730702968	-1.613966284	0.256004283
piRNA-19544	4.536239237	1.445615654	-1.649812695	0.256024048
piRNA-16240	0.916792002	3.286018275	1.841674141	0.261134736
piRNA-15149	1.501869855	4.610270417	1.618091574	0.264495561
piRNA-00753	4.505377773	10.36825612	1.202453276	0.271506977

piRNA-20365	11.4245136	4.520072216	-1.337715017	0.272492483
piRNA-04801	3.847146082	1.382354474	-1.476661002	0.273925519
piRNA-19951	0.507920413	1.59906289	1.654552319	0.274939824
piRNA-17216	0.756280014	0.207114495	-1.868491972	0.278176035
piRNA-19167	46.36006877	15.47295276	-1.583134169	0.296885545
piRNA-20813	19.57306748	8.261922404	-1.244320457	0.31122989
piRNA-12734	0.532194159	0.160418882	-1.730108712	0.312131072
piRNA-13322	0.126114946	0.407944776	1.693634604	0.318552407
piRNA-04308	6.803204781	13.7312721	1.013178866	0.322845231
piRNA-19179	0.122830282	0.387202105	1.656420514	0.327990365
piRNA-03239	0.122830282	0.387202105	1.656420514	0.327990365
piRNA-19143	0.122830282	0.387202105	1.656420514	0.327990365
piRNA-09857	0.122830282	0.387202105	1.656420514	0.327990365
piRNA-01929	0.122830282	0.387202105	1.656420514	0.327990365
piRNA-14626	0.122830282	0.387202105	1.656420514	0.327990365
piRNA-13175	0.122830282	0.387202105	1.656420514	0.327990365
piRNA-17183	0.122830282	0.387202105	1.656420514	0.327990365
piRNA-13028	0.122830282	0.387202105	1.656420514	0.327990365
piRNA-17608	0.122830282	0.387202105	1.656420514	0.327990365
piRNA-18467	0.122830282	0.387202105	1.656420514	0.327990365
piRNA-12935	0.122830282	0.387202105	1.656420514	0.327990365
piRNA-20815	0.947933808	2.667231287	1.492484705	0.337264247

piRNA-16963	4.986942071	11.92792753	1.258116047	0.340925371
piRNA-19949	0.391211626	0.128971574	-1.600896121	0.349958748
piRNA-19241	0.413228337	0.140862096	-1.552655739	0.358995776
piRNA-20492	29.23231417	59.61273091	1.028056422	0.368672766
piRNA-16796	0.613364013	1.778408375	1.535771219	0.369290606
piRNA-16742	1253.241112	528.9357057	-1.24449973	0.377826192
piRNA-17033	8.886625125	19.79591763	1.155495409	0.377919678
piRNA-20541	38.43700824	78.58943165	1.031839271	0.3780547
piRNA-16792	5.22804525	2.6935574	-0.956758819	0.378792552
piRNA-11968	0.113902015	0.307585165	1.433192649	0.387775711
piRNA-03810	0.113902015	0.307585165	1.433192649	0.387775711
piRNA-06557	0.113902015	0.307585165	1.433192649	0.387775711
piRNA-19169	3.717795546	1.433186951	-1.375220621	0.388374184
piRNA-04800	0.626526785	1.738503721	1.472398063	0.389515629
piRNA-04920	0.356155879	0.131195559	-1.440789921	0.390142948
piRNA-18084	0.356155879	0.131195559	-1.440789921	0.390142948
piRNA-01101	4.479385076	2.375906311	-0.914822747	0.40750167
piRNA-20364	21.55444407	11.53125575	-0.902435722	0.407763201
piRNA-14923	5.713196989	2.510760974	-1.186173585	0.414213246
piRNA-20362	12.75112515	32.17664151	1.335389195	0.415242842
piRNA-20490	11.51350056	6.353012402	-0.857813797	0.417370151
piRNA-02158	16.38623386	7.810831121	-1.068936337	0.42591891

piRNA-13545	0.343225204	0.13847205	-1.309560688	0.429572095
piRNA-20363	3.94381237	9.061079212	1.200091975	0.430762431
piRNA-16980	118.6397344	67.18165025	-0.820448134	0.432289607
piRNA-05660	6.200263934	12.72862804	1.037675391	0.432675863
piRNA-04993	7.366424327	12.94139601	0.812956843	0.448221055
piRNA-13624	13.28814481	27.80641911	1.065278267	0.448939475
piRNA-10894	11.00826783	6.246124512	-0.817554242	0.450268819
piRNA-17724	2.609504721	4.600737387	0.818089097	0.456569567
piRNA-20466	6.803379502	3.522561392	-0.949626717	0.464013384
piRNA-01168	6.317133123	3.454134371	-0.870945768	0.467547689
piRNA-08982	1.369900901	3.127467414	1.19092332	0.469043583
piRNA-09228	26.15345224	13.73912616	-0.928711146	0.469357382
piRNA-11410	0.223388403	0.101512555	-1.137896118	0.47979011
piRNA-20910	0.084512947	0.182516914	1.110785896	0.480887029
piRNA-06245	0.084512947	0.182516914	1.110785896	0.480887029
piRNA-08927	0.241031263	0.110611578	-1.123717881	0.485273874
piRNA-20009	10.02614337	6.009037607	-0.738560913	0.485632968
piRNA-19822	13.72159068	8.641883823	-0.667029994	0.488604359
piRNA-03674	0.081929646	0.173086548	1.079036117	0.490535275
piRNA-05919	0.081929646	0.173086548	1.079036117	0.490535275
piRNA-14636	0.081929646	0.173086548	1.079036117	0.490535275
piRNA-17936	14.99487291	23.42807729	0.643769264	0.493985646

piRNA-04987	34.75975008	18.83157731	-0.884263866	0.496921928
piRNA-19224	10.49419477	6.245821776	-0.748628164	0.501624647
piRNA-20979	0.078038992	0.159388117	1.030277022	0.505524169
piRNA-16926	27.09436234	16.41579617	-0.722907971	0.51776071
piRNA-19368	7.522742596	4.720436242	-0.672338534	0.521379363
piRNA-20582	4.295147969	7.306523398	0.766477271	0.52205902
piRNA-00801	9.545895174	5.865170676	-0.702707403	0.56411398
piRNA-01179	1.235644525	0.630934418	-0.969701806	0.573512938
piRNA-04152	84.4005829	137.4748708	0.703843063	0.574462675
piRNA-07979	2.900255419	1.68577156	-0.782770912	0.584549976
piRNA-15150	3.119872079	5.449453513	0.804624683	0.595023801
piRNA-01318	7.161731243	11.6022499	0.696024314	0.60151646
piRNA-19201	3.106120131	4.891294541	0.655102715	0.602761369
piRNA-20619	9.047224664	5.64732461	-0.679907737	0.611088426
piRNA-00291	4.433856833	7.335748492	0.726381989	0.611638851
piRNA-17184	4.296969472	7.030313278	0.710269451	0.613509585
piRNA-18800	1.421106603	2.584232178	0.862720912	0.614813513
piRNA-19675	4.876313254	3.284714738	-0.570022723	0.614915805
piRNA-16271	0.100598464	0.061852015	-0.701715771	0.622000323
piRNA-12925	0.100598464	0.061852015	-0.701715771	0.622000323
piRNA-19324	4.236121472	6.15796689	0.539710151	0.622986094
piRNA-12681	3.940264504	2.436443113	-0.69351594	0.646152921

piRNA-00330	8.094972775	5.100732766	-0.66632171	0.657851037
piRNA-00805	19.47089598	26.9800827	0.470575498	0.67195839
piRNA-16931	2.914363527	4.751872282	0.705315216	0.673719474
piRNA-16735	10.73104664	14.6447877	0.448596485	0.675140111
piRNA-03728	6.752604878	5.084414808	-0.409362406	0.676552295
piRNA-20828	2.167713942	3.090724758	0.511770795	0.678340023
piRNA-04506	21.44476281	29.66015656	0.467900854	0.685087555
piRNA-19911	3.290230016	4.534640861	0.462799851	0.6854546
piRNA-05018	0.395126285	0.633019929	0.679937098	0.689192792
piRNA-08033	2.968468744	1.886171015	-0.654258435	0.702362219
piRNA-17104	6.245843842	8.943637985	0.517965293	0.703084799
piRNA-19574	3.605829243	5.186832094	0.52452259	0.706316616
piRNA-19752	52.25138525	75.41840045	0.529447267	0.709354802
piRNA-08921	1.090070801	1.658437061	0.60540242	0.724288265
piRNA-16945	8.453162563	10.53078897	0.317050428	0.735124826
piRNA-19354	16.52795593	22.76064195	0.461632936	0.736977084
piRNA-18570	4.890658477	3.800070833	-0.364002412	0.749097555
piRNA-09895	1.380894579	0.943943696	-0.548830471	0.749136397
piRNA-17061	8.956649856	11.38396912	0.34597254	0.756843355
piRNA-08112	1.858947399	2.6486613	0.510777421	0.757883561
piRNA-20450	6.027825356	8.008873711	0.409961752	0.757895517
piRNA-20668	2.13936734	2.826024011	0.401589501	0.764762031

piRNA-18849	0.967778275	1.374571282	0.506233266	0.768004007
piRNA-01042	0.599026201	0.424407154	-0.497170134	0.769549
piRNA-20548	8.603348795	7.034170135	-0.290518099	0.771072309
piRNA-17723	9.28077457	11.42493097	0.299868326	0.779346743
piRNA-08983	6.092703825	7.748898261	0.346908592	0.78683901
piRNA-18292	5.0857279	6.249999945	0.297401901	0.787691544
piRNA-19068	1.590906058	2.160639813	0.441609941	0.790556968
piRNA-17716	3.308205942	4.009365254	0.277324805	0.799744044
piRNA-13306	313.6362882	261.9811265	-0.259629609	0.80871869
piRNA-00441	11.11771201	9.162306917	-0.279077121	0.81219713
piRNA-16970	11.47331048	10.00841405	-0.197068343	0.812409421
piRNA-20829	2.584583274	3.069446499	0.248046838	0.825586574
piRNA-19628	10.21088538	8.160389884	-0.32339798	0.835508559
piRNA-04150	5130.679183	4141.111062	-0.309131923	0.841078627
piRNA-10155	0.990362806	1.24811267	0.333719137	0.846107333
piRNA-19823	105.5228654	122.6812021	0.217358562	0.846706165
piRNA-14634	1.099655229	0.880472485	-0.320701446	0.85212504
piRNA-19914	3.44266431	2.996300051	-0.200343409	0.852690533
piRNA-01107	2.054595815	2.485740445	0.27482105	0.856971444
piRNA-01311	312.8206734	368.5093363	0.236365312	0.870816365
piRNA-18573	6.460810667	5.66513041	-0.189606031	0.87969524
piRNA-00560	5.17893951	5.850382892	0.17587434	0.890876939

piRNA-07635	5.42696518	6.006612629	0.146405974	0.896682509
piRNA-09295	7.954251772	7.262164613	-0.131326596	0.896971527
piRNA-09502	2.611384173	2.273994841	-0.199585734	0.904085567
piRNA-01207	10.9736657	12.1129464	0.142504303	0.905135982
piRNA-20575	2.032394105	1.796796201	-0.177753402	0.908547025
piRNA-00651	8.933511057	9.696738318	0.118272255	0.911742408
piRNA-20814	7.704337856	7.051809727	-0.127677422	0.913128877
piRNA-12753	12.66906493	11.33574243	-0.160431164	0.914489687
piRNA-00552	6.189487257	6.670125012	0.107893901	0.92014081
piRNA-00586	76.69355028	70.57441396	-0.119960012	0.92649382
piRNA-19676	74.51705593	68.71533484	-0.116938583	0.927717119
piRNA-18165	1.456260161	1.31963067	-0.142133902	0.928896398
piRNA-16659	7.348242035	6.96987444	-0.07626648	0.931917917
piRNA-17194	1.389814785	1.259872837	-0.141614508	0.934242304
piRNA-09011	1.214490954	1.120611169	-0.116065968	0.944672962
piRNA-02485	2.409763667	2.247717325	-0.100431051	0.951965664
piRNA-16946	9.159254846	9.681307348	0.079971648	0.954987224
piRNA-04918	1.38426103	1.336114359	-0.051072523	0.976243387
piRNA-19102	2.514790311	2.434950867	-0.046545448	0.976632025
piRNA-16975	2.581494498	2.64731468	0.036323172	0.979088466
piRNA-00329	3.169369284	3.26176108	0.041455343	0.979287443
piRNA-09051	3.530130267	3.592150661	0.025126438	0.983909279

piRNA-22628	1.229361995	1.250097339	0.024130645	0.985370808
piRNA-16984	0.949777509	0.962804418	0.019653168	0.989816629
piRNA-01184	4.49698504	4.451840888	-0.014556054	0.992801178
piRNA-20401	2.846394012	2.827728786	-0.009491626	0.995483135
piRNA-16828	25.44256254	25.43651969	-0.000342694	0.999777434

N: Normal; C: Cancer.
